# Identification of the Bovine *CSN3* Core Promoter Region and the Relationships Between *CSN3* Promoter Polymorphisms and the *CSN3* A and B Alleles

**DOI:** 10.3390/ani15020134

**Published:** 2025-01-08

**Authors:** Wenqing Li, Xiaoyang Wang, Xiuyang Xu, Pinhui Wu, Tong Fu, Liyang Zhang, Tengyun Gao

**Affiliations:** 1 College of Life Science, Henan Agricultural University, Zhengzhou 450046, China; liwenqing605@163.com (W.L.); wangxiaoyang312@163.com (X.W.); xxy15713631303@163.com (X.X.); phwu2022@163.com (P.W.); 2 College of Animal Science and Technology, Henan Agricultural University, Zhengzhou 450046, China; futong2004@126.com

**Keywords:** *CSN3*, promoter, polymorphism, allele

## Abstract

Reportedly, the number of κ-casein (κ-CN) B alleles increases the proportion of κ-CN to total protein and the κ-CN content. This phenomenon is caused by single-nucleotide polymorphisms (SNPs) in the promoter region of *CSN3*, which encodes the B variant. Therefore, a series of 5′-deleted promoter plasmids were constructed to define the core promoter of *CSN3*. The promoter activity was analyzed by comparing the luciferase activity among the recombinant vectors with truncated promoters. No mutation occurred in the core promoter region (5′-ctatcgtcagatctttcctttctgtcatcttcctattggtg-3′) of *CSN3* in 40 cows. A 2092 bp promoter region of *CSN3* was re-sequenced for detection, and nine variants were found, of which only three variants had mutation frequencies > 40%, which were −1002T>−, −1654T>A, and −2039T>G. The *CSN3* promoter polymorphisms did not correlate with the *CSN3* A and B alleles according to the Pearson’s chi-square test (*p* > 0.05). Moreover, the luciferase activity analysis of the *CSN3* promoter showed no difference among pGL3 recombinants with different polymorphic *CSN3* promoters (*p* > 0.05). In the genetic selection of dairy cows, mutations in the *CSN3* core promoter should be focused upon. These findings provide a reference for the regulatory mechanism of bovine milk proteins and offer guidance for the genetic selection and breeding of cows.

## Simple Summary

κ-casein (κ-CN) A and B are the most common protein variants. Many studies have reported that κ-CN B is more favorable than κ-CN A in increasing the content of protein, casein, and κ-casein in milk. It was speculated that this difference may be due to the polymorphism of the κ-CN promoter. No base mutations were detected in the core promoter region of κ-CN. There were two main types of polymorphisms in the κ-CN promoter region, but these two types of promoter polymorphisms were not related to the A and B variants of κ-CN, and there was no significant difference in the activity of the two types of promoter polymorphism.

## 1. Introduction

Milk protein genotypes are closely associated with its composition and quality [1,2,3]. Casein (CN) is the main protein in milk, accounting for 80% of the protein in milk, and is composed of αs1-CN, αs2-CN, β-CN, and κ-CN [4]. In recent years, the A2-like β-CN variant has been studied more due to the influence of consumer demand [5]. It has been found that the A2-like β-CN variant is correlated with higher estimated breeding values for the milk protein yield and lower estimated breeding values for the milk protein percentage [6]. As a crucial component in maintaining the emulsion stability of milk, the κ-CN genotype significantly affects milk’s composition and coagulation traits [7,8,9]. Among the 13 protein mutations detected in κ-CN, κ-CN A and B are the most common protein variants [1,10]. The differences between the A and B variants lie in amino acid positions 136 and 148. The amino acids at positions 136 and 148 of the A variant correspond to threonine (Thr) and L-aspartic acid (Asp), respectively, whereas the B variant corresponds to L-isoleucine acid (Ile) and alanine (Ala), respectively [4]. Amino acid mutations in CSN3 affect milk’s coagulation properties.

In addition to the above impacts, the genetic variant κ-CN B is associated with increased protein, casein, and κ-CN contents [11,12,13,14]. Polymorphisms in the noncoding regions of milk protein genes affect protein transcription [15,16]. For example, one SNP at the 1847th (T/C) bp in the noncoding region of the α-lactalbumin gene correlated with protein contents in Chinese Holstein dairy cows [17].

Research on *CSN3* polymorphisms has focused mainly on the coding regions, whereas noncoding region polymorphisms have rarely been reported. A single-nucleotide polymorphism (SNP) in the second intron of *CSN3* is associated with several milk production traits [18]. At present, the limited studies on noncoding region polymorphisms have only conducted association analyses with the production performance or bioinformatic predictions, and almost none have performed functional verification [18,19]. Robitaille studied the polymorphism of the 5′-flanking region of *CSN3* and detected three mutations at the −999 (>T), −1651 (A>T), and −2035 (G>T) sites upstream of the transcription initiation site [20]. However, they reported that no SNP was associated with the allele-specific accumulation of *CSN3* mRNA.

Many studies have shown that low concentrations of κ-CN in milk are associated with both κ-CN A and κ-CN E, whereas high concentrations of κ-CN are associated with κ-CN B; the reasons for these observations have not been elucidated [13,20,21]. Therefore, we hypothesized that polymorphisms of the κ-CN promoter were correlated with κ-CN mutants and caused differences in the expression of κ-CN mutants. This study will provide a reference for the regulatory mechanism of κ-CN A and B mutations and provide guidance for the genetic selection of dairy cows.

## 2. Materials and Methods

### 2.1. Animals, Samples, and Promoter Analysis

**Figure 1:**
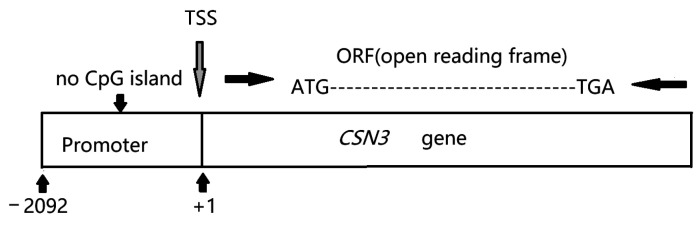
The scheme of the *CSN3* gene promoter (2092 bp upstream of the TSS).

Forty healthy Chinese Holstein cows were provided by Qi County Farm in Kaifeng, Henan Province, China. Most of the cows were in mid-lactation, and they were housed in a free-stall barn, fed a total mixed ration (TMR), and milked three times daily (8:00 a.m., 3:00 p.m., and 8:00 p.m.). Samples were only taken once at the regular milking time of 3:00 p.m. Two 15 mL milk samples were collected from each cow at the regular milking times. Each sample was centrifuged at 2000× *g* for 10 min at 4 °C to isolate the fat in the upper layer and milk whey in the middle layer to obtain somatic cells. Somatic cells from one 15 mL sample were stored at −20 °C for subsequent DNA extraction, and the somatic cells from the second 15 mL sample were mixed with 1 mL of TRIzol, transferred into a 1.5 mL RNase-free centrifuge tube, and stored at −80 °C for subsequent RNA extraction and coding region sequencing. Based on certain references and an alignment analysis, we used 2092 bp sequences upstream of the transcription start site (TSS) as the possible promoter sequence for *CSN3* [20,22]. MethPrimer software (http://www.urogene.org/methprimer/, accessed on 21 October 2024) was used to predict the presence of CpG islands in the promoter region of the *CSN3* gene. No CpG islands were detected in the promoter region of the *CSN3* gene (Figure 1).

### 2.2. Construction of a Progressively Shortened pGL3-Basic Recombinant Vector

Genomic DNA was extracted from a milk somatic cell sample according to the manufacturer’s instructions (TIANGEN, DP304, Beijing, China). With the use of Primer BLAST on the NCBI platform and online software (https://crm.vazyme.com/cetool/simple.html, accessed on 21 October 2024), homology arm primers for the *CSN3* promoter and the pGL3-Basic reporter gene vector were designed. Moreover, the downstream primer CSN3-P1R was fixed, and six upstream primers were designed for gradual truncation. The primer sequences were designed based on the reference sequence (NC_037333.1) and are listed in Table 1. Using genomic DNA as a template, the promoter region of bovine *CSN3* was amplified using a high-fidelity PCR kit (Takara, R045A, Dalian, China). The reaction system and conditions were as per the instructions provided by the manufacturer. PCR products of the correct length were ligated with the linearized pGL3-Basic vector according to the instructions of the ClonExpress II One Step Cloning Kit (Vazyme, C112-02, Nanjing, China). These constructs were transformed into Top10 competent cells (TIANGEN, CB104-02, Beijing, China) via heat shock according to the product handbook. The transformants were incubated at 37 °C overnight on selective LB agar plates containing 100 μg/mL ampicillin. Single colonies were selected to verify that the correct target fragment had been amplified using PCR. Plasmids were extracted from the bacterial cultures of single colonies using the TIANprep Plasmid Midi Kit (TIANGEN, DP106, Beijing, China). The constructed recombinant vector was verified using HindIII digestion (Supplementary Figures S1 and S2). The recombinant vector was sent to Tsingke Biotech Co., Ltd. (Beijing, China) for sequencing. The correct sequence alignment indicated that the recombinant vector had successfully been constructed. The six recombinant vectors were named pCSN3-2000/+59, pCSN3-1618/+59, pCSN3-1172/+59, pCSN3-744/+59, pCSN3-426/+59, and pCSN3-162/+59. As there was no significant differences in the activity of the six CSN3 promoter truncations described above, four additional promoter truncations (primers in Table 2) were constructed using pCSN3-221/+59 as a template, and the recombinant vectors were named pCSN3-122/+59, pCSN3-82/+59, pCSN3-42/+59, and pCSN3-2/+59.

### 2.3. Cell Transfection and the Dual-Luciferase Reporter Assay

First, 293T cells were seeded into 24-well plates at a density of 1 × 10^5^ cells/well in high-glucose DMEM (HyClone, SH30022, Logan City, UT, USA) supplemented with 10% fetal bovine serum (Cat. No. 11011-8611). According to the instructions provided for Lipofectamine^TM^ 3000 (Thermo Fisher Scientific, L3000008, Shanghai, China), 0.8 µg/well of recombinant plasmid and 0.05 µg/well of the internal reference plasmid pRL-TK were co-transfected into the 293T cells 24 h later. The empty pGL3-Basic vector served as the negative control. After 48 h of transfection, the cells were lysed according to the instructions provided with the Dual-Luciferase Reporter Gene Assay Kit (Beyotime, RG027, Shanghai, China). The relative luciferase activity was detected using a chemiluminescent plate reader (SpectraMax, Shanghai, China). The ratio of firefly luciferase to Renilla luciferase (F/R) was calculated to determine the core promoter region of the *CSN3* gene. All of the reporter assays were repeated 6 times.

### 2.4. Sequencing the Core Promoter Region of Bovine CSN3

DNA was extracted from 40 milk somatic cells using a Genomic DNA Kit (TIANGEN, DP304, Beijing, China). The core promoter region of the *CSN3* gene was amplified using the original primers (CSN3-P5F and CSN3-P1R), which amplified a 485 bp fragment. The PCR system included 2 μL of DNA template, 25 μL of ApexHF HS DNA Polymerase FS Master Mix (Accurate, AG12202, Changsha, China), 2 μL of upstream primer (CSN3-P5F), 2 μL of downstream primer (CSN3-P1R), and 19 μL of RNase-free H_2_O. The amplification conditions were predenaturation at 94 °C for 1 min, denaturation at 98 °C for 10 s, annealing at 55 °C for 15 s, and elongation at 72 °C for 20 s for a total of 30 cycles, followed by elongation at 72 °C for 2 min. The PCR products from 25 cattle were pooled into one sample, and those from 50 cattle were pooled into two samples and sent to Tsingke Biotech Co., Ltd. (Beijing, China) for two-way sequencing. The sequencing results were aligned using DNAStar software (7.1 version).

### 2.5. Promoter Mutations Outside the CSN3 Core Promoter Region

The 2092 bp promoter fragments of 40 samples were amplified using DNA extracted from the milk somatic cells as a template. The upstream primer sequence was 5′-AGTTCAGTATATTTGATATGG-3’, and the downstream primer sequence was 5′-AGTTGGCCTTTCCACTGTAAG-3′. PCR was performed according to the manufacturer’s instructions provided with the kit (Accurate Cat. AG12301, China). The PCR products were ligated into the pGM-T vector according to the manufacturer’s instructions (Tiangen, cat. VT302-02, China). The plasmids were transfected into competent TOP10 cells. After clone screening and amplification, plasmids were extracted and sequenced. The sequencing results were aligned with a reference sequence (NC_037333.1) using online software (https://www.novopro.cn/tools/muscle.html, accessed on 1 June 2023), and the SNPs were analyzed with reference to the variant table in Ensemble.

### 2.6. Identification of the CSN3 A and B Alleles and Their Relationships with CSN3 Promoter Polymorphisms

RNA was isolated from each sample using TRIzol reagent (Invitrogen, Carlsbad, CA, USA) according to the manufacturer’s instructions. Total RNA extracts were employed to synthesize cDNA using a PrimeScript™ One Step RT–PCR Kit (TaKaRa, cat. RR055A Otsu, Japan). cDNA was used as a template to amplify the full-length (846 bp) *CSN3*. The upstream primer *CSN3* cDNA-F was 5′-CTTACAGTGGAAAGGCCAAC-3′, and the downstream primer *CSN3* cDNA-R was 5′-TGCATTTGATTGGCTTTATT-3′. PCR amplification was performed according to the instructions and conditions provided in the kit (Accurate, cat. AG12301, China). The PCR products were sent to Shanghai Biological Engineering Co., Ltd. (Shanghai, China) for two-way sequencing, and the sequencing results were analyzed using DNAStar. CSN3 variants were analyzed according to the nomenclature of the milk proteins [23].

Samples with two *CSN3* promoter polymorphisms (15 samples with no base mutant promoters and 14 samples with three base mutant promoters) were selected and analyzed for correlation with the *CSN3* genotypes.

### 2.7. Construction of the Promoter Mutation Recombinant Vector and the Dual-Luciferase Reporter Assay

Based on the promoter sequencing results, one sample without base mutations (sample no. 26) and one sample with three high-frequency mutant bases (sample no. 36) were selected as the two DNA templates for constructing two recombinant vectors. The homology arm primers were redesigned. The upstream primer sequence was 5′-atctgcgatctaagtaagcttAGTTCAGTATATTTGATATGG-3′, and the downstream primer sequence was 5′-cagtaccggaatgccaagcttAGTTGGCCTT TCCACTGTAAG-3′. The methods used for target gene amplification and vector construction are described in the section on pGL3-Basic recombinant vector construction. The two recombinant vectors were named pCSN3-no-base mutation and pCSN3-three-base mutation, respectively.

### 2.8. Statistical Analysis

Comparisons between multiple groups were performed using a one-way analysis of variance (ANOVA) in the comparison means procedure using SPSS version 22. Comparisons between the two groups were performed using the independent samples *t* test in the comparison means procedure in SPSS version 22. The results are presented as the means ± standard error of the figures. A statistical *p* value was set with 0.05 indicating statistical significance. Correlations between the *CSN3* promoter polymorphisms and *CSN3* genotypes were analyzed using the Pearson’s chi-square test under cross-tabulation in the descriptive statistics procedure using SPSS version 22.

## 3. Results

### 3.1. Identification of the Bovine CSN3 Core Promoter

**Figure 2:**
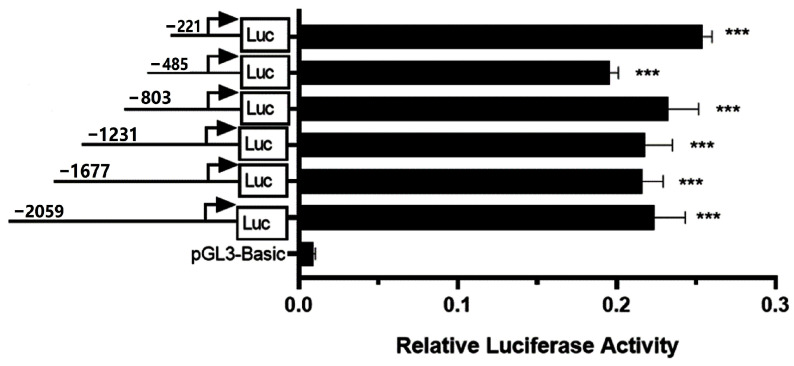
Relative luciferase activities of six recombinant vectors with a truncated *CSN3* promoter (162 bp to 2000 bp upstream of the TSS). The progressively truncated promoter fragments are depicted on the left, and promoter activity is indicated on the right. Three asterisks indicate that the relative luciferase activity of each recombinant was very significantly different from that of pGL3-Basic (negative control, *p* < 0.001). However, no significant difference was observed among the six recombinants (*p* > 0.05).

**Figure 3:**
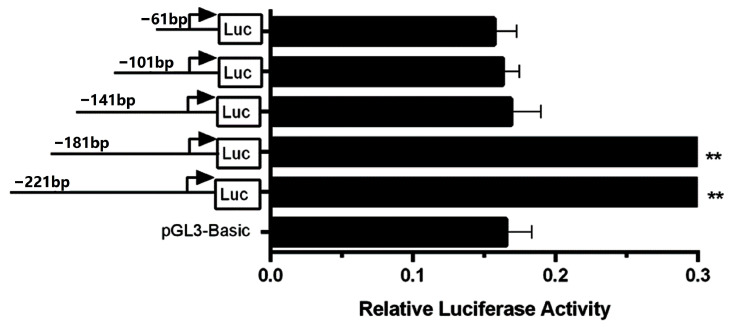
Relative luciferase activities of five recombinant vectors with a truncated *CSN3* promoter (2 bp to 162 bp upstream of the TSS). The progressively truncated promoter fragments are depicted on the left, and promoter activity is indicated on the right. Two asterisks indicate that the relative luciferase activities of pCSN3 −162/+59 (−221 bp) and pCSN3 −122/+59 (−181 bp) (*p* < 0.01) were significantly greater than those of pGL3-Basic, pCSN3 −82/+59 (−141 bp), pCSN3 −42/+59 (−101 bp), and pCSN3 −2/+59 (−61 bp) (*p* < 0.01). There were no significant differences between pGL3-Basic and pCSN3 −82/+59 (−141 bp), pCSN3 −42/+59 (−101 bp), or pCSN3 −2/+59 (−61 bp) (*p* > 0.05).

No CpG islands were found in the promoter region of the *CSN3* gene. As shown in Figure 2, the promoter activities of the six recombinant vectors were significantly greater than those of pGL3-Basic (*p* < 0.001); however, there were no significant differences among the six recombinant vectors (*p* > 0.05). These results suggest that there was no core promoter region between 162 and 2000 bp upstream of the TSS. Four shortened recombinant vectors were constructed using pCSN3-221/+59 as a template, and the relative luciferase activity was detected, as shown in Figure 3. When the promoter length was shortened from 221 to 181 bp, the relative luciferase activity of the recombinant vector did not significantly change (*p* > 0.05). However, when the promoter was shortened further to 141 bp, the relative luciferase activity decreased significantly (*p* < 0.01), indicating there was a core promoter region between 141 bp and 181 bp (between 82 bp and 122 bp upstream of the TSS). This region plays an important role in the transcriptional activation of the *CSN3* gene. When the promoter was shortened from 141 bp, the decreased promoter activity did not recover. The core promoter sequence of the bovine *CSN3* gene was determined to be 5′-ctatcgtcagatctttcctttctgtcatcttcctattggtg-3′.

### 3.2. Sequence Variations in the Bovine CSN3 Core Promoter Region

**Figure 4:**
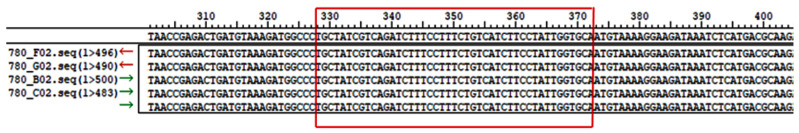
Sequence alignment of the core promoter region of *CSN3*. The last sequence is the reference sequence (NC_037333.1). The red arrows represent reverse PCR sequencing, and the green arrows represent forward PCR sequencing. The bases in the red box represent the core promoter region.

The sequencing results of the *CSN3* core promoter region are shown in Figure 4, which indicate that there was no base mutation in the core promoter region of bovine *CSN3*.

### 3.3. CSN3 Promoter Polymorphisms

In the 2092 bp *CSN3* promoter region, nine variants were detected after removing the variant that occurred only once, and four of them had SNP IDs (Table 3). Among the four SNP loci, 641 bp upstream of the TSS were recorded at AT>− in Ensemble, whereas A>− was detected with a low frequency (5%) in this study. The three loci with higher mutation frequencies were T>− (−1002, rs136772334, 45%), T>A (−1654, rs110303451, 42.5%), and T>G (−2039, rs109870556, 47.5%). No base mutations were detected in the core promoter region (between 82 and 122 bp upstream of the TSS) of *CSN3*.

Of the samples collected, 15/40 had no base mutations, and 14/40 had more than three high-frequency mutations. In the subsequent dual-luciferase reporter assay, two types of polymorphic samples (those with no base mutations and those with three high-frequency mutations) were selected for constructing the recombinant vector to detect the activities of the two types of promoters. The mutation combinations in the other samples were singular, with a frequency of only 1/40 (the total number of samples was 40). Except for sample no. 14, all three mutant bases in the other samples were based on the above three high-frequency mutant bases, such as those in samples no. 13 and 21.

### 3.4. Identification of CSN3*A and CSN3*B Variants and Their Relationships with CSN3 Promoter Polymorphisms

**Figure 5:**
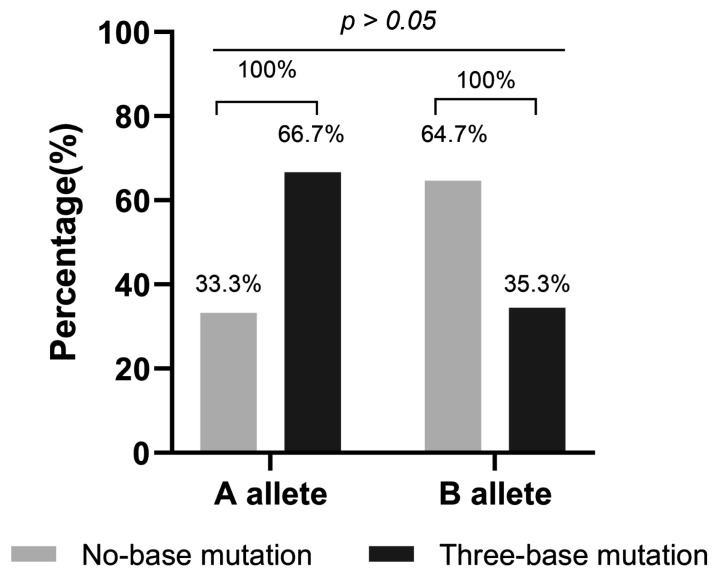
Percentages of two *CSN3* promoter polymorphisms in the *CSN3* A and B allele samples. The results of the statistical analysis were obtained from 15 samples with promoters with no base mutations (nos. 2, 3, 9, 11, 12, 16, 20, 25, 26, 27, 28, 31, 33, 34, and 39) and 14 samples with promoters with three base mutations (nos. 1, 4, 6, 7, 8, 17, 22, 23, 24, 29, 30, 32, 36, and 38). A Pearson’s chi-square test revealed that there was no significant correlation between the *CSN3* A or B alleles and *CSN3* promoter polymorphisms (*p* > 0.05).

The results of *CSN3* transcript sequencing for the 40 samples revealed that only two variants, CSN3*A and CSN3*B, were present. The distributions of CSN3*A and CSN3*B in the samples are presented in Table 4. Pearson’s chi-square test was used to examine the relationship between the *CSN3* genotypes and *CSN3* promoter polymorphisms. As shown in Figure 5, samples with three base mutant promoters accounted for more of the *CSN3* A allele, and samples with no base mutant promoter accounted for more of the *CSN3* B allele. However, there was no significant correlation between the *CSN3* A or B alleles and *CSN3* promoter polymorphisms (*p* > 0.05).

### 3.5. Differences in the Promoter Activities of CSN3

**Figure 6:**
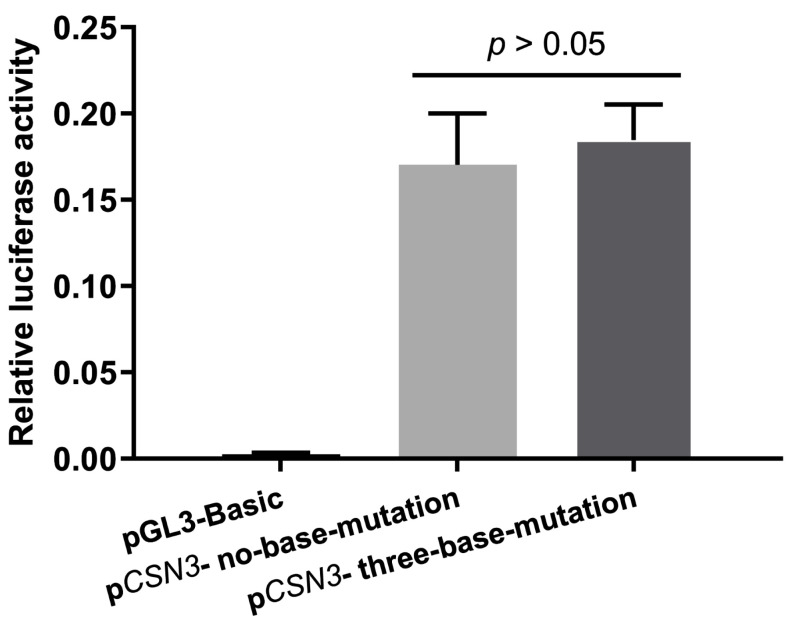
Relative fluorescence ratios of the *CSN3* promoter in the two recombinant vectors. pGL3-Basic was used as a negative control. “*p* > 0.05” indicates no significant difference in the promoter activity between the two recombinant vectors.

A dual-luciferase reporter assay was performed to determine whether the two types of promoter mutations affected the promoter activity. The relative fluorescence ratios of the two recombinant vectors, p*CSN3*-no-base-mutation and p*CSN3*-three-base-mutation, were not significantly different, as shown in Figure 6 (*p* > 0.05).

## 4. Discussion

The core promoter is considered a short sequence flanking the TSS, which is sufficient to assemble the RNA polymerase II transcription machinery and initiate transcription [24]. Because base changes in the core promoter region may have a greater impact on the gene expression, we first detected base mutations in the core promoter region. The core promoter is usually located between −50 bp and −80/−100 bp upstream of the TSS [25]. In this study, the core promoter region of Chinese Holstein *CSN3* was found to be located in the −82–−122 region upstream of the TSS, which was consistent with the above findings. Although many canonical core promoter elements have been identified, many genes do not contain these elements [25]. Moreover, there are mixed and matched modules in the core promoter; different combinations of the core promoter elements may support the assembly of distinct components of the transcription machinery and the recruitment of RNA Pol II, allowing for the expression of tissue-specific and/or developmentally regulated genes [26]. Consequently, the core promoter regions of specific genes must be confirmed experimentally. The base of the *CSN3* core promoter region did not change in any of the samples in this study, further verifying the conservation of the core promoter region. However, farmers in dairy cattle genetic selection must pay close attention to mutations in the *CSN3* core promoter, which can have a significant impact on the amount of κ-CN in milk.

In this study, three high-frequency mutations were detected in the upstream region of the *CSN3* gene outside the core promoter: −1002T >− (rs136772334), −1654T>A (rs110303451) and −2039T>G (rs109870556). This result was consistent with the mutation reported by Robitaille, but the mutation position differed by three to four bases [20]. The main reason for this difference was that the present mutation was compared with the sequence of κ-CN B as the reference sequence (κ-CN B is the mutation-free reference sequence in the Ensemble database), whereas Robitaille used the sequence of κ-CN A as the reference sequence. Mutations at these three sites occurred simultaneously in most of our samples, representing a haplotype, whereas some of the other samples had one or fewer mutations relative to these three loci. Our study revealed that approximately two-thirds of the κ-CN A samples had promoters with three base mutations, and similarly, approximately two-thirds of the κ-CN B samples had promoters with no base mutations. In agreement with Robitaille’s report, there was no significant association between the *CSN3* promoter polymorphism and the κ-CN A and B alleles [20]. Thus, it can be inferred that differences in the κ-CN A and B contents in milk are not caused by polymorphism in the CSN3 promoter. Does the *CSN3* promoter cause differences in expression activity due to these two polymorphisms? Our dual-fluorescence assay further confirmed that there was no difference in the expression activity between the two promoters. Therefore, the base change in the *CSN3* promoter detected by the current data cannot be used as the screening basis for increasing milk protein or κ-CN content in Chinese Holstein cow breeding.

Whether the *CSN3* A and B alleles are associated with 3′-flanking region polymorphisms and whether the differences in their expression levels are due to 3′-flanking region polymorphisms need to be confirmed further.

## 5. Conclusions

To the best of our knowledge, this is the first study to report the core promoter region of the *CSN3* gene. All of the base mutations detected in the promoter region of *CSN3* were outside the core promoter region. Base mutations in the core promoter of *CSN3* may have significant effects on the expression of *CSN3*, so they should be particularly considered in the future genetic selection of dairy cows. The *CSN3* A and B alleles were not significantly associated with *CSN3* promoter polymorphisms, and *CSN3* promoter polymorphisms did not cause significant differences in promoter activity.

## Figures and Tables

**Table 1:** Primers for the promoter and truncated promoter fragments of the bovine *CSN3* gene.

Primer Name	Primer Sequences (5′-3′)	Size (bp)	Positions Relative to the TSS
CSN3-P1F	atctgcgatctaagtaagcttACCAGAGCAACAATTTTTGCCT	2059	−2000/+59
CSN3-P2F	atctgcgatctaagtaagcttCTCCTGCCCCACTGTAGAA	1677	−1618/+59
CSN3-P3F	atctgcgatctaagtaagcttCATGACCATCACTCTGAACATTC	1231	−1172/+59
CSN3-P4F	atctgcgatctaagtaagcttTGTGAAGAAAGGGGAATCCTC	803	−744/+59
CSN3-P5F	atctgcgatctaagtaagcttCAACCACAGCCCATAATATATGTAG	485	−426/+59
CSN3-P6F	atctgcgatctaagtaagcttCTGCATTCCATTAACCGAGAC	221	−162/+59
CSN3-P-R	cagtaccggaatgccaagcttTTCCTTGTGACCGTCAGCT		

Note: Lowercase letters represent homologs, where aagctt is the restriction site of HindIII, and uppercase letters represent primer sequences of the target gene. TSS refers to the transcription start site. These results were also applied to the primers listed in Table 2.

**Table 2:** Further truncated primers for the bovine CSN3-P6 promoter.

Primer Name	Primer Sequences (5′-3′)	Size (bp)	Positions Relative to the TSS
CSN3-P6F	atctgcgatctaagtaagcttCTGCATTCCATTAACCGAGACTG	221	−162/+9
CSN3-P7F	atctgcgatctaagtaagcttCTATCGTCAGATCTTTCCTTTCTGTC	181	−122/+59
CSN3-P8F	atctgcgatctaagtaagcttGCAATGTAAAAGGAAGATAAATCTCATG	141	−82/+59
CSN3-P9F	atctgcgatctaagtaagcttAACACCCTTTAATTAGTCTCTGGTTATT	101	−42/+59
CSN3-P10F	atctgcgatctaagtaagcttTCCTTACAGTGGAAAGGCCAAC	61	−2/+59
CSN3-P-R	cagtaccggaatgccaagcttTTCCTTGTGACCGTCAGCTCTT		

**Table 3:** Mutant bases and mutation frequencies of the *CSN3* promoter in 40 cattle.

	Allele Position Before The TSS and SNP ID	−66 A>G No SNP ID	−394 T>A No SNP ID	−641 A>− rs134762502(AT>−)	−982 A>T No SNP ID	−1002 T>− rs136772334	−1269 T>C No SNP ID	−1654 T>A rs110303451	−1867 T>A No SNP ID	−2039 T>G rs109870556
Number of Mutant Bases (Sample Number) and Mutation Frequencies										
No base mutation (Nos. 2, 3, 9, 11, 12, 16, 20, 25, 26, 27, 28, 31, 33, 34, and 39)										
One base mutation (no. 5)							C			
One base mutation (no. 10)			A							
One base mutation (no. 19)										G
One base mutation (no. 37)				—						
Two base mutations (no. 15)					T	—				
Two base mutations (no. 18)					T				A	
Two base mutations (no. 35)								A		G
Two base mutations (no. 40)						—				G
Three base mutations (nos. 1, 4, 6, 7, 8, 17, 22, 23, 24, 29, 30, 32, 36, and 38)						—		A		G
Three base mutations (no. 14)		G	A						A	
Four base mutations (no. 13)		G				—		A		G
Five base mutations (no. 21)				—		—	C	A		G
Mutation frequencies		5%	5%	5%	5%	45%	5%	42.5%	5%	47.5%

Note: NC_037333.1 was used as the reference sequence. The numbers in parentheses represent the sample numbers of 40 cattle, and samples with consistent sequencing results were merged into the same row. After the sequencing analysis, mutations that appeared only once in all samples were regarded as representative of the absence of mutations. Base deletions were classified as base mutation types. The last row of the table shows the mutation frequency of each base (number of mutant base samples/total number of samples). The three high-frequency bases and their mutation frequencies were highlighted.

**Table 4:** Distributions of CSN3*A and CSN3*B in the samples.

Allele	CSN3*A	CSN3*B
Sample numbers	4, 5, 7, 8, 10, 13, 14, 17, 20, 21, 22, 24, 27, 28, 29, 30, 34, 37, 40	1, 2, 3, 6, 9, 11, 12, 15, 16, 18, 19, 23, 25, 26, 31, 32, 33, 35, 36, 38, 39

## Data Availability

The original contributions presented in this study are included in the article and Supplementary Material, and further inquiries can be directed to the corresponding authors.

## Supplementary Materials

The following supporting information can be downloaded at https://www.mdpi.com/article/10.3390/ani15020134/s1. Figure S1 shows the HindIII restriction maps of the six truncated promoter recombinant vectors. M indicates the presence of DNA markers. Lanes 1, 3, 5, 7, 9, and 11 show six truncated promoter recombinant vectors, and lanes 2, 4, 6, 8, 10, and 12 show the two fragments of the recombinant vector formed after HindIII digestion. Figure S2 shows the HindIII restriction map of the four additional truncated promoter recombinant vectors. M indicates the presence of DNA markers. Lanes 1, 3, 5, 7, 9, 11, 13, and 15 show the additional truncated promoter recombinant vectors, whereas lanes 2, 4, 6, 8, 10, 12, 14, and 16 show the two fragments of the recombinant vector formed after HindIII digestion.
