# Supplementary material for: Identification of the Bovine CSN3 Core Promoter Region and the Relationships Between CSN3 Promoter Polymorphisms and the CSN3 A and B Alleles
Source: Animals (Basel). 2025 Jan 8;15(2):134. doi: 10.3390/ani15020134 (PMC11758600; doi:10.3390/ani15020134)
Supplement: Supplementary file 1 [file animals-15-00134-s001.zip › animals-3374990-supplementary.pdf]

Supplementary materials

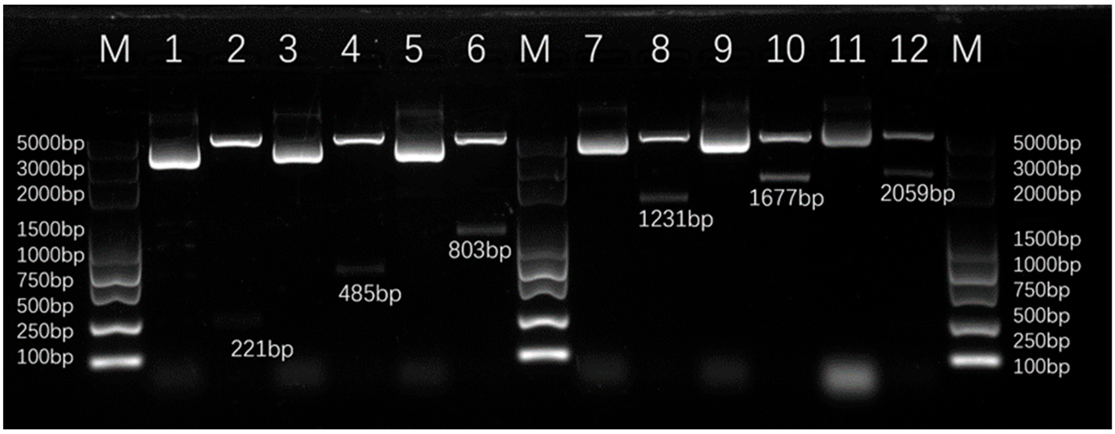

Figure S1. The HindIII restriction maps of the six truncated promoter recombinant vectors. M indicates the presence of DNA markers. Lanes 1, 3, 5, 7, 9, and 11 show six truncated promoter recombinant vectors and; lanes 2, 4, 6, 8, 10, and 12 show the two fragments of the recombinant vector formed after HindIII digestion.

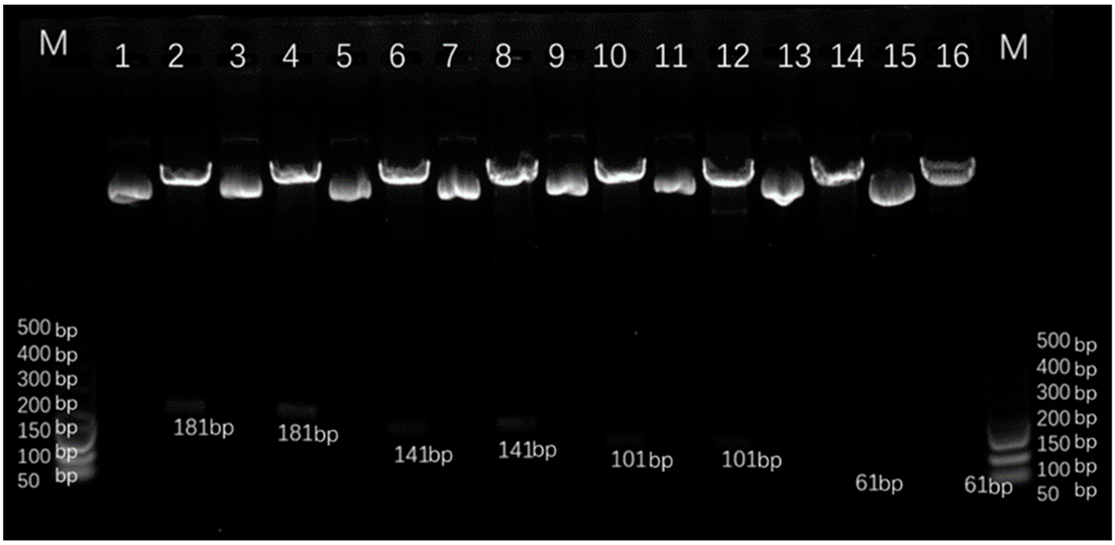

Figure S2. The HindIII restriction map of the four further truncated promoter recombinant vectors. M indicates the presence of DNA markers. Lanes 1, 3, 5, 7, 9, 11, 13, and 15 show the additional truncated promoter recombinant vectors; whereas

lanes 2, 4, 6, 8, 10, 12, 14, and 16 show the two fragments of the recombinant vector formed after HindIII digestion.
